# Modern contraceptive use among women in need of family planning in India: an analysis of the inequalities related to the mix of methods used

**DOI:** 10.1186/s12978-021-01220-w

**Published:** 2021-08-21

**Authors:** Fernanda Ewerling, Lotus McDougal, Anita Raj, Leonardo Z. Ferreira, Cauane Blumenberg, Divya Parmar, Aluisio J. D. Barros

**Affiliations:** 1 International Center for Equity in Health, Universidade Federal de Pelotas, Pelotas, Brazil; 2 Center on Gender Equity and Health, University of California San Diego, San Diego, USA; 3 Postgraduate Program in Epidemiology, Universidade Federal de Pelotas, Pelotas, Brazil; 4 King’s Centre for Global Health and Health Partnerships, School of Population Health and Environmental Sciences, King’s College London, London, UK; 5 Marechal Deodoro 1160, 3rd floor, Pelotas, RS Brazil

**Keywords:** Family planning, Contraceptive use, Health inequalities

## Abstract

**Objective:**

To evaluate the type of contraceptives used by women in need of family planning in India and the inequalities associated with that use according to women's age, education, wealth, subnational region of residence and empowerment level.

**Methods:**

Using data from the Indian National Family and Health Survey-4 (2015–2016), we evaluated the proportion of partnered women aged 15–49 years with demand for family planning satisfied (DFPS) with modern contraceptive methods. We also explored the share of each type of contraception [short- (e.g., condom, pill) and long-acting (i.e., IUD) reversible contraceptives and permanent methods] and related inequalities.

**Results:**

The majority (71.8%; 95% CI 71.4–72.2) of women in need of contraception were using a modern method, most (76.1%) in the form of female sterilization. Condom and contraceptive pill were the second and third most frequently used methods (11.8% and 8.5%, respectively); only 3.2% reported IUD. There was a nearly linear exchange from short-acting to permanent contraceptive methods as women aged. Women in the poorest wealth quintile had DFPS with modern methods at least 10 percentage points lower than other women. We observed wide geographic variation in DFPS with modern contraceptives, ranging from 23.6% (95% CI 22.1–25.2) in Manipur to 93.6% (95% CI 92.8–94.3) in Andhra Pradesh. Women with more accepting attitudes towards domestic violence and lower levels of social independence had higher DFPS with modern methods but also had higher reliance on permanent methods. Among sterilized women, 43.2% (95% CI 42.7–43.7) were sterilized before age 25, 61.5% (95% CI 61.0–62.1) received monetary compensation for sterilization, and 20.8% (95% CI 20.3–21.3) were not informed that sterilization prevented future pregnancies.

**Conclusion:**

Indian family planning policy should prioritize women-centered care, making reversible contraceptive methods widely available and promoted.

**Supplementary Information:**

The online version contains supplementary material available at 10.1186/s12978-021-01220-w.

## Plain Language Summary

Ensuring universal access to sexual and reproductive health and reproductive rights for all women is one of the Sustainable Development Goals, promoted by the United Nations and adopted by 193 countries, including India. To address women’s need for contraception, the provision of a wide range of safe, effective and affordable contraceptive methods is essential. In this study, we evaluated the type of contraceptives used by women in need of contraception in India and the inequalities in contraceptive use among different subgroups of women. The majority (71.8%) of women in need of contraception were using a modern method, most (76.1%) in the form of female sterilization. Condom and contraceptive pill were the second and third most frequently used methods (11.8% and 8.5%, respectively); only 3.2% reported IUD. We observed an exchange from short-acting to permanent contraceptive methods as women aged. The poorest women presented demand for family planning satisfied (DFPS) with modern methods at least 10 percentage points lower than other women. There was wide geographic variation in DFPS with modern contraceptives, ranging from 23.6% in Manipur to 93.6% in Andhra Pradesh. Women with more accepting attitudes towards domestic violence and lower levels of social independence had higher DFPS with modern methods but also had higher reliance on permanent methods. Among sterilized women, 43.2% were sterilized before age 25, 61.5% received monetary compensation for sterilization, and 20.8% were not informed that sterilization prevented future pregnancies. Indian family planning policy should prioritize women-centered care, making reversible contraceptive methods widely available and promoted.

**Supplementary Information:**

The online version contains supplementary material available at 10.1186/s12978-021-01220-w.

## Introduction

Ensuring universal access to sexual and reproductive health and reproductive rights for all women is Target 5.6 of the Sustainable Development Goals, promoted by the United Nations and adopted by 193 countries. To address women’s need for family planning, the provision of a wide range of safe, effective and affordable contraceptive methods is essential [1]. The mix of methods offered must cater to women’s needs and preferences. It is also important to note that every contraceptive method has advantages and disadvantages [2]. Thus, it is essential that women are fully informed about them so they can make an informed decision on which method is more appropriate for their specific situation. A nationally representative study found that India's demand for family planning satisfied (DFPS) with modern contraceptive methods was 70% in 2005, with heavy reliance on female sterilization rather than reversible contraceptive methods [3]. These findings already suggested the need for greater focus on improving access to reversible methods, especially for women who wish to delay or space pregnancies but are not ready to commit to ending their fertility.

Family planning policies in India have historically been aimed at controlling population growth rather than advancing women's reproductive rights and choices [4, 5]. This led to an explicit promotion of sterilization, targeted nearly exclusively towards women. Government policy has since changed, as laid out in the 2014 Family Planning 2020 action plan [6], which still promotes sterilization with monetary compensation (both for individuals undergoing the procedure and for the health providers) but also includes reversible modern contraception methods. At this time, three new contraceptive methods were introduced in the National Family Planning program—injectable contraceptive, a non-hormonal weekly pill and progesterone-only pills for lactating mothers—all provided free-of cost. Intrauterine device (IUD) remains low despite being covered under public health services for decades and condom use only increased subsequent to HIV prevention efforts in the country. However, despite these changes in the policy environment, the use of reversible contraceptive methods, as well as male sterilization, is still low in India, and female sterilization continues to be the dominant method, accounting for two-thirds of the total contraceptive use [1, 7].

In this paper, we evaluated the coverage of DFPS with modern methods in India and the share of each type of contraception (long-acting reversible, short-acting reversible and permanent methods) being used. Additionally, we assessed inequalities in these indicators according to women's age, education, wealth, subnational region of residence and empowerment level.

## Methods

We used data from the National Family and Health Survey (NFHS-4) conducted in India in 2015–2016. This survey was implemented using a multistage sampling strategy. In the first stage, 28,586 primary sampling units (PSU) were selected with a probability of selection proportional to the PSU size. The selected PSUs were mapped, and their households were listed. In the second stage, 22 households from each PSU were systematically selected, totaling 628,892 households included in the sample. All women aged 15–49 years who slept in the selected households the night before the interview were invited to participate in the survey. In total, 699,686 women were interviewed on topics including family planning. An extended version of the questionnaire was applied to a subsample of 112,351 women, including, among others, questions related to women's empowerment and domestic violence. This subsample is representative at state-level, while the larger sample is representative at district level [8].

Our main outcome of interest is DFPS with modern methods among women aged 15–49 years who are currently married or in union (hereafter referred to as partnered women). This indicator is defined as the proportion of women using a modern contraceptive method among those in need of contraception (women who are fecund and do not want to become pregnant within the next 2 years, or who are unsure about whether or when they want to become pregnant) [9]. Women are considered infecund if they (1) are married for five years or more, did not use contraception and had not gotten pregnant in that period; (2) reported that they cannot get pregnant; (3) reported menopause, hysterectomy or never menstruated; or (4) had last period more than 6 months ago and are not postpartum amenorrheic. According to the indicator definition, pregnant women with a mistimed or unwanted pregnancy are also considered in need of family planning [9]. We used the definition of modern contraception proposed by Hubacher and Trussel [10], that classifies modern contraceptive methods as technological products or medical procedures that affect natural reproduction. According to this definition, the following contraceptive methods were considered as modern: contraceptive pills, condoms (male and female), IUD, sterilization (male and female), injectables, diaphragms, spermicidal agents (foam/jelly), and emergency contraception. The modern contraceptive methods analyzed were also classified as: (1) short-acting reversible contraception (SARC), including contraceptive pills, injectables, condoms, diaphragms, spermicidal agents and emergency contraception; (2) long-acting reversible contraception (LARC), including solely IUD; and (3) permanent methods, comprised of male and female sterilization. Hormone implants (LARC) and patches are also considered modern methods but were recorded in the DHS questionnaire as “other modern methods”. However, none of the women interviewed reported this category.

We evaluated the proportion of partnered women who have DFPS with modern methods and the share of each type of contraceptive among the users. To better understand the patterns among subgroups, we also stratified the analyses by women's age, education (categorized as none; primary; secondary or higher), household wealth quintiles, based on the asset index included in the survey dataset (Q1 being the poorest and Q5 the richest quintile), subnational region and women's empowerment as measured by the SWPER global index [11]. The SWPER global index is an individual-level indicator based on 14 questions that allows the assessment of three empowerment domains: (1) attitude to domestic violence, which comprises questions related to the women's opinion on whether beating the wife is justified in five situations (if the woman goes out without telling the husband; neglects the children; argues with her husband; refuse to have sex; and if she burns the food); (2) social independence, that includes the women's access to information, educational attainment, age at first marriage and first child, and the difference in age and education between the woman and her husband; and (3) decision making, which is comprised of three questions on who makes decisions in the household in regard to the respondent's health care, major expenses and visits to family and relatives. The SWPER is a cross-culturally tested tool that allows the measurement of women's empowerment at individual-level and among subgroups of women. The resulting scores are categorized as low, medium, and high empowerment level using the cut-offs provided with the SWPER global methodology. Full details on the construction of the index and its validity are presented elsewhere [11].

Given the extremely high DFPS with permanent methods in the country, we further explored the available descriptive information about the women that were sterilized, including: age and parity at sterilization, whether they were told that sterilization would mean no more children, whether they received monetary compensation to undergo the procedure (and the average compensation received) according to wealth quintiles, and whether they regret the sterilization according to their parity at sterilization. These analyses were also restricted to currently partnered women, but the results including all women, regardless of their marital status, were virtually the same (results not shown).

All estimates were calculated taking the survey sample design (including clusters, strata, and sample weights) into account. All analyses were conducted using the statistical software Stata 15 (StataCorp. 2017. *Stata Statistical Software: Release 15*. College Station, TX: StataCorp LLC). NFHS-4 is a publicly available source of data, so ethical clearance was not required for this study. The NFHS-4 study protocol, including all the survey questionnaires, was approved by the International Institute for Population Sciences Institutional Review Board and the ICF Institutional Review Board.

## Results

Among the 339,540 partnered women aged 15–49 years in need of contraception, 71.8% (95% CI 71.4–72.2) were using a modern contraceptive method. Table 1 presents the share of contraceptive methods used by women with DFPS with a modern method, showing that most women using contraceptives in India rely on permanent contraception (76.1%, 95% CI 75.5–76.6), while 20.7% (95% CI 20.3–21.2) are using SARC, and 3.2% (95% CI 3.1–3.3) LARC methods. Analyzing the specific method being used, results show that 75.5% (95% CI 75.0–76.0) of the women using a modern method are sterilized, while only 0.6% (95% CI 0.5–0.6) had male partners who were subjected to a vasectomy. Condom and contraceptive pill were the most used methods after sterilization, being respectively used by 11.8% (95% CI 11.5–12.1) and 8.5% (95% CI 8.2–8.9) of the women and thus comprising almost all of SARC use. Only 3.2% (95% CI 3.1–3.3) relied on LARC (i.e., IUD).

**Table 1:** Type of contraception being used among partnered women aged 15–49 years currently using contraception in India, 2015–2016 (N = 243,814)

	%	95% CI
**Short-acting reversible contraception**	20.7	20.3–21.2
Pill	8.5	8.2–8.9
Injectables	0.4	0.3–0.4
Condoms	11.8	11.5–12.1
**Long-acting reversible contraception**	3.2	3.1–3.3
IUD	3.2	3.1–3.3
**Permanent contraception**	76.1	75.5–76.6
Female sterilization	75.5	75.0–76.0
Male sterilization	0.6	0.5–0.6

Other short-acting reversible contraceptive methods, such as diaphragm, spermicidal agents (foam/jelly), and emergency contraception were reported by only 9 women (< 0.01%), thus they were not included in the table

**Fig. 1:**
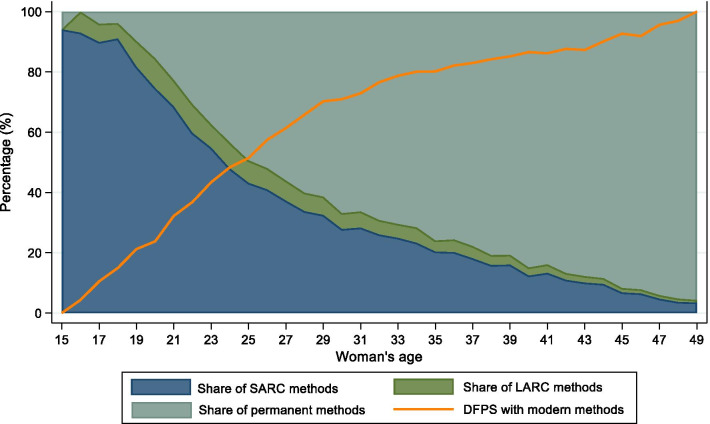
Demand for family planning satisfied (DFPS) with modern methods and the share of each type of contraceptive method among partnered women aged 15–49 currently using contraception of modern contraception and according to women's age. India, 2015–2016 (N = 339,540). *Note* Short-acting reversible contraceptive (SARC) methods include condoms (male and female), injectables, diaphragms, jelly or foam and emergency contraception; Long-acting reversible contraceptive (LARC) methods include intrauterine devices (IUD); and Permanent methods include male and female sterilization

Large disparities in the proportion of partnered women with DFPS with modern methods were observed according to the women's age, with higher coverage among older women (Fig. 1). Women aged 40 years or older presented over three times the DFPS with modern methods when compared to women aged 15 to 19 years of age. Among women using modern methods, there is nearly a linear exchange of short-acting to permanent contraceptive methods as age increases. In the early twenties, around 20% of the users rely on permanent methods; this share quickly rises and reaches 80% in the forties. The share of LARC is relatively constant at around 5% in women of 15 to 35 years old, slowly decreasing until the end of reproductive age.

**Fig. 2:**
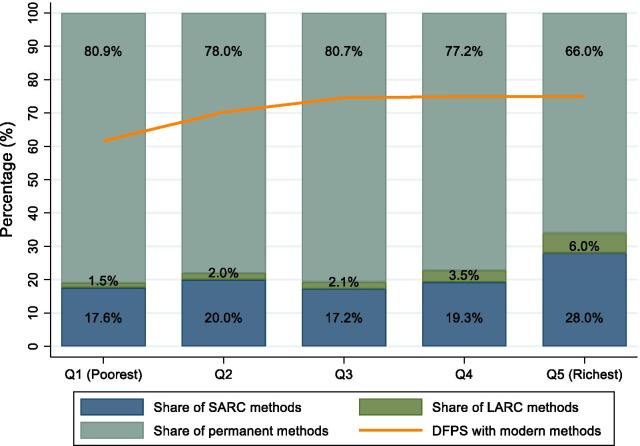
Demand for family planning satisfied (DFPS) with modern methods and the share of each type of contraceptive being used among partnered women aged 15–49 currently using contraception according to household wealth quintiles. India, 2015–2016 (N = 339,540). *Note* Short-acting reversible contraceptive (SARC) methods include condoms (male and female), injectables, diaphragms, jelly or foam and emergency contraception; Long-acting reversible contraceptive (LARC) methods include intrauterine devices (IUD); and Permanent methods include male and female sterilization

**Fig. 3:**
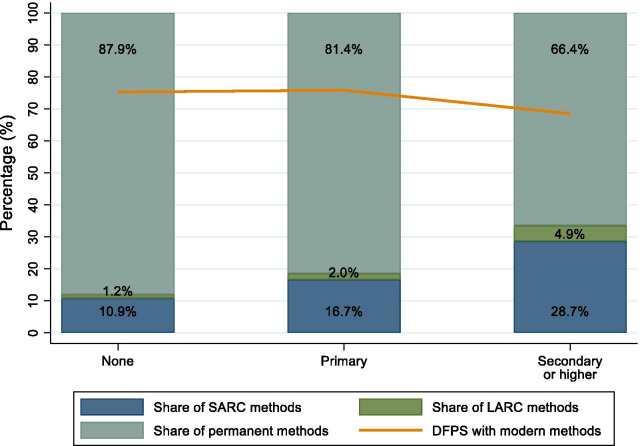
Demand for family planning satisfied (DFPS) with modern methods and the share of each type of contraceptive being used among partnered women aged 15–49 currently using contraception according to women's education. India, 2015–2016 (N = 339,540). *Note* Short-acting reversible contraceptive (SARC) methods include condoms (male and female), injectables, diaphragms, jelly or foam and emergency contraception; Long-acting reversible contraceptive (LARC) methods include intrauterine devices (IUD); and Permanent methods include male and female sterilization

Across household wealth quintiles, women in the poorest quintile lagged behind the rest, with approximately 10% points lower DFPS, as observed in Fig. 2. Within the richest quintile, there was also a larger share of SARC and LARC methods and a lower reliance on permanent contraception. Figure 3 shows that more educated women had around 10 percentage points lower coverage of DFPS with modern methods when compared to less educated women. However, the share of SARC and LARC methods was much higher among more educated women, while less-educated women more frequently use permanent methods, revealing once again the extreme reliance on sterilization for satisfying family planning needs.

**Fig. 4:**
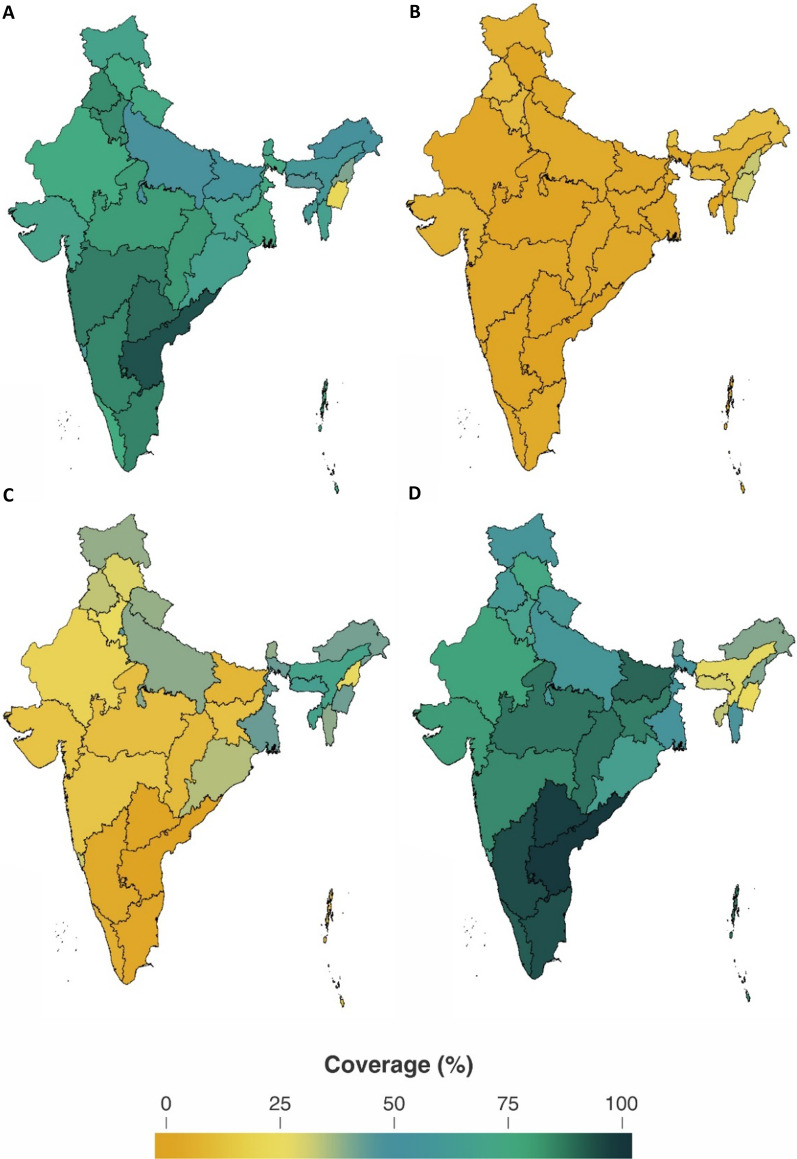
Maps of **A** demand for family planning satisfied (DFPS) with modern methods, **B** share of long-acting reversible contraceptives; **C** share of short-acting reversible contraceptives; and **D** share of permanent contraceptive methods among partnered women aged 15–49 in India, 2015–2016 (N = 339,540). *Note* Short-acting reversible contraceptive (SARC) methods include condoms (male and female), injectables, diaphragms, jelly or foam and emergency contraception; Long-acting reversible contraceptive (LARC) methods include intrauterine devices (IUD); and Permanent methods include male and female sterilization

Strong geographic variation can be clearly observed in DFPS with modern methods (Fig. 4A), ranging from 23.6% (95% CI 22.1–25.2) in Manipur to 93.6% (95% CI 92.8–94.3) in Andhra Pradesh. Southern states, except for Lakshadweep archipelago, present higher coverage when compared to northeastern states and also the highest shares of permanent methods. Generally, reliance on permanent contraception was remarkably high, accounting for over 70% of modern contraception in 17 out of the 36 Indian states/union territories (Fig. 4D). The share of short-acting contraceptive use only surpasses 50% in Assam, Chandigarh, Meghalaya and Tripura (Fig. 4B). Long-lasting share fails to reach 5% in 22 states, and only exceeds 15% in Manipur and Nagaland (Fig. 4C). Estimates for all states and their confidence intervals are presented in the Additional file 1: Table S1.

**Fig. 5:**
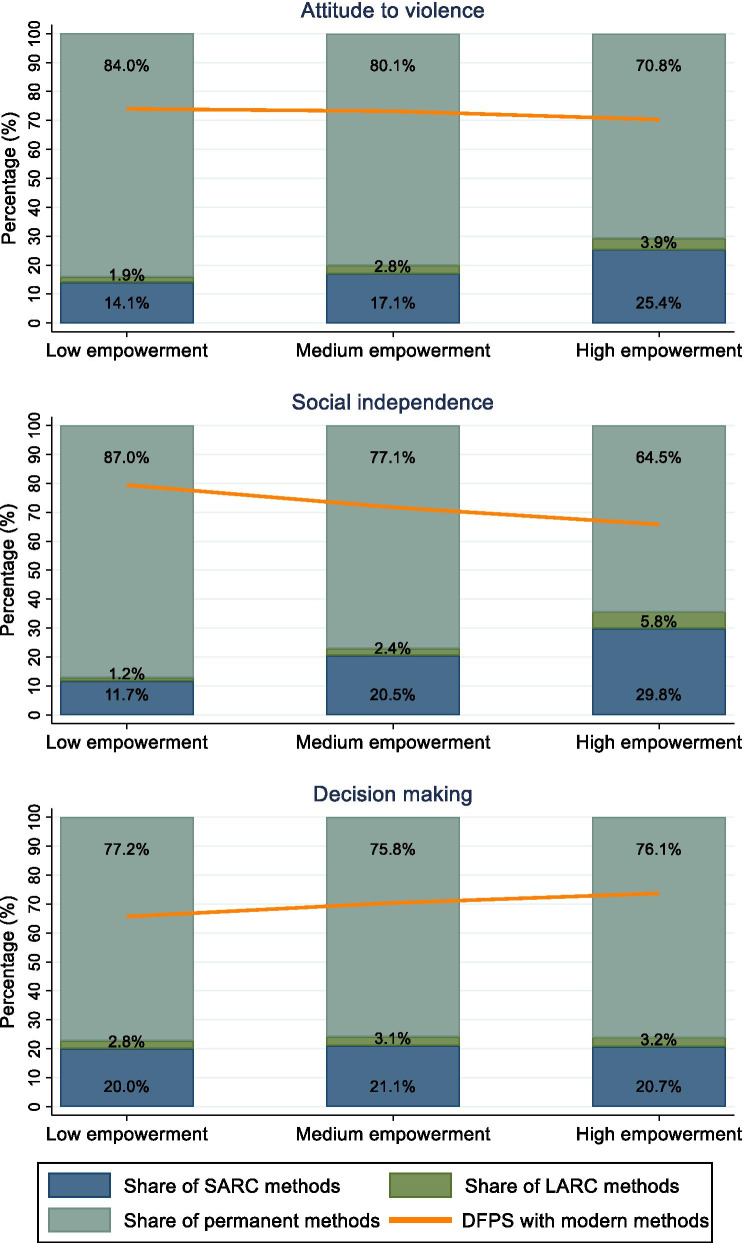
Demand for family planning satisfied (DFPS) with modern methods and the share of each type of contraceptive being used among partnered women aged 15–49 currently using contraception according to the women's empowerment level in attitude to violence, social independence, and decision-making domains. India, 2015–2016 (N = 59,434). *Note* Short-acting reversible contraceptive (SARC) methods include condoms (male and female), injectables, diaphragms, jelly or foam and emergency contraception; Long-acting reversible contraceptive (LARC) methods include intrauterine devices (IUD); and Permanent methods include male and female sterilization

According to the empowerment level (using the sub-sample of women), Fig. 5 shows that women who were more empowered in terms of attitude to violence and social independence domains of the SWPER index had lower DFPS with permanent methods, but higher with SARC methods. Regarding the DFPS with permanent methods, there was a 13.2 percentage point gap between the low vs. highly empowered women in attitude to violence (84.0% vs 70.8%, respectively) and a 22.5 percentage point gap in the social independence domain (87.0% vs 64.5%, respectively). Even though DFPS was lower among more empowered women in these domains, they presented a higher share of SARC and LARC methods, while less empowered women more frequently relied on permanent methods. The decision-making domain showed different patterns, with higher DFPS with permanent methods among the highly empowered women, though the difference was not as marked (77.2%, 95% CI 75.4–78.9 vs 76.1%, 95% CI 75.1–77.1) for the low and high empowerment women, respectively). For decision-making, the share of each type of contraceptive used was very similar regardless of the women’s empowerment level.

Table 2 shows a description of the women that have undergone sterilization. It shows that 42.9% (95% CI 42.4–43.4) of the sterilized women had the procedure before they were aged 25 years old. The vast majority of the women had 2 or more children at time of sterilization; only 3.3%, 95% CI 3.1–3.5 had none or one child at sterilization. One in five sterilized women (20.8%, 95% CI 20.3–21.2) were not told that sterilization would mean no more children, with no difference according to the time since sterilization (results not shown). Even so, the percentage of women who said they regretted sterilization was a modest 6.9% (95% CI 6.7–7.2), ranging from 5.8% (95% CI 5.5–6.1) among those that had 4 or more children when the procedure was done to 11.8% (95% CI 10.3–13.1) among women that had up to one child. Most (61.2%; 95% CI 61.0–62.1) of the sterilized women said they received monetary compensation for the sterilization, ranging from 76.3% (95% CI 75.6–77.1) among the poorest women to 40.9% (95% CI 39.7–42.2) among the richest. The average amount of monetary compensation received was higher among richer women. There was no substantial difference in the proportion of women receiving compensation according to the time since the procedure was done (results not shown).

**Table 2:** Description of partnered women aged 15–49 years that were sterilized. India, 2015–2016 (N = 185,429)

	%	95% CI
Age at sterilization		
< 25 years	42.9	42.4–43.4
25–29 years	35.4	35.0–35.8
30–34 years	15.7	15.5–16.0
> 35 years	6.0	5.8–6.2
Parity at sterilization		
0–1 child	3.3	3.1–3.5
2 children	43.4	42.8–43.9
3 children	29.4	29.0–29.7
4 + children	24.0	23.6–24.4
Not told sterilization would mean no more children	20.8	20.3–21.2
Regret sterilization	6.9	6.7–7.2
Regret according to parity at sterilization		
0–1 child	11.8	10.3–13.1
2 children	7.5	7.2–7.9
3 children	6.5	6.1–6.8
4 + children	5.8	5.5–6.1
Received monetary compensation for sterilization	61.2	61.0–62.1
Received monetary compensation by wealth quintiles		
Q1 (Poorest)	76.3	75.6–77.1
Q2	71.4	70.6–72.1
Q3	66.1	65.3–66.9
Q4	57.2	56.3–58.1
Q5 (Richest)	40.9	39.7–42.2

Average monetary compensation by wealth quintiles	Rupees (₹)	95% CI
Q1 (Poorest)	659.3	639.3–679.4
Q2	637.0	617.9–656.0
Q3	692.2	668.3–716.1
Q4	737.0	702.6–771.5
Q5 (Richest)	784.0	727.7–840.3

## Discussion

Findings from this study demonstrate that nearly three in every four (72%) partnered women in need of contraception in India are using a modern method. This is a negligible improvement from DFPS observed in 2005 data, which was at 70% [3]. Further, three-quarters of this use is represented by female sterilization, again demonstrating inadequate progress in the promotion of reversible contraceptive use in the nation in the past decade. Further, 43% of sterilized women are under 25 years of age, with the fastest increase in sterilization prevalence occurring between ages 19–25 years [8]. This directly corresponds with India's median age at first birth of 21 years, median birth interval of 32 months, and total fertility rate of 2.2 children [8]. India's reliance on female sterilization is largely due to a historical legacy of government policy that promoted sterilization to control population growth [12, 13], as well as patriarchal norms that view vasectomy as a threat to masculinity and sexuality [14, 15]. Importantly, there is some indication of increasing modern contraceptive use based on preliminary state level data collected in 2019–2020, under the NFHS-5, although sterilization continues to be the dominant form of contraceptive used [16].

Reliance on SARC or LARC was much less common (20.7% and 3.2%, respectively). Several factors likely contribute to these low shares. There is an ongoing fear of side effects and health issues associated with the use of different SARC and LARC methods [17]. Additionally, research on contraceptive use in India suggests that familial pressure, as well as gender and social norms, play a strong role and that these norms have shifted little over time [17–19]. Many women do not use contraception following marriage in order to demonstrate their fertility, and as a result of pressure from husbands, in-laws and communities [20–22]. Deviation from these norms may facilitate greater uptake and sustained use of these methods, thus reducing reliance on sterilization. Our results showed that the highest share of SARC and LARC use were found among women with higher socioeconomic status, education and empowerment levels. Even though these subgroups of women presented higher reliance on non-permanent methods, they have lower DFPS with modern methods, which reinforces the difficulty of using contraception that is not sterilization. After infrequent sex, the most common reasons women reported for not using contraception in India were the opposition of the husband or someone else (19.7%), lack of access (9.8%), fatalistic approach (9.1%), respondent opposition (8.1%) and health concerns (7.4%) [19]. Across age groups, LARC use was highly invariant. However, at younger ages, there is a much higher share of SARC methods, that is almost linearly exchanged to permanent methods as women get older. Evidence shows that, even among sexually active unpartnered women, sterilization is the most commonly used contraceptive method in India [8]. We may, however, see a change when new data become available due to the *Mission Parivar Vikas*, a government initiative launched in 2016 to promote modern contraceptives in 146 high fertility districts via financial incentives for women and family planning providers (financial incentives apply for injectable contraception, IUD and sterilization) [23].

There is evidence to suggest that women from more marginalized backgrounds achieve higher levels of family and community status, as well as greater freedom of movement, only following their sterilization [24]. For these women, therefore, current social structures may impede their ability to access some of the rights enjoyed by wealthier, more educated and more empowered women prior to sterilization. These findings highlight the need not only for more targeted efforts to support access and uptake of SARC and LARC methods among more socially vulnerable women, but also a need to understand in greater detail how normative, structural and economic barriers may affect their contraceptive decision-making. Our results also offer caution regarding sterilization, however. One in five sterilized women were not told that their procedures meant that their childbearing would be complete, and well more than half (62%) received financial incentives for undergoing sterilization. In the context of a long history of forced and coercive sterilizations [12], in which sterilization targets and financial incentives for women who undergo sterilization and health workers who enable those procedures [25] still exist, these results indicate a need for a greater understanding of women's information, choices and autonomy regarding these procedures. Sterilization regret, while a concern at any level, was very low in this sample (7%). However, given the large proportion of women undergoing sterilization, the number of women experiencing regret are considerable [26]. We estimate that more than 92 million women in reproductive age are sterilized in India, thus around 6.5 million women regret having undergone the procedure. Additionally, sterilization levels were similar across wealth quintiles, indicating that incentivization alone is not driving this high prevalence. Given the substantial geographic heterogeneity in the prevalence of demand for family planning satisfied with permanent methods, with higher coverage in the south, central and west of India, and lower coverage in the north and east, in line with previous research, additional geospatial analyses may be warranted [20].

Importantly, this study was able to identify the ways women meet their need for family planning across both types of contraception and domains of empowerment. The least socially independent women (e.g. those with lower levels of information access, educational attainment, and age at first marriage and childbirth, and with higher gaps in age and education between spouses) have the highest reliance on permanent methods. These findings correspond with extensive research from India, consistently showing greater uptake of sterilization and younger age at sterilization among socially marginalized relative to more privileged women [8, 13, 24, 27–29].

This study has some limitations. Data were derived from a self-reported survey and are thus subject to recall bias. Causality cannot be inferred from this observational, cross-sectional analysis. Due to the unique history of contraceptive uptake in India, as well as the current highly skewed method mix, results may have limited generalizability beyond India. Also, we know that contraceptive use has increased since 2015–16, when these data were collected, based on preliminary findings from state level data collected in 2019–2020 [16]. However, the current analyses use the only nationally representative data available from India, and more recent data show similar patterns of contraceptive use as seen in these 2015–16 data, albeit at higher rates.

## Conclusions

This study adds to the growing body of research aimed at explaining the relationship between women's empowerment and contraceptive use. It is clear that empowerment affects the methods with which women satisfy their need for family planning, with women with more gender-equitable views on violence against women and more socially independent being less likely to use permanent methods and more likely to use SARC and LARC methods than their less empowered counterparts. There is a need to make LARC and SARC methods more widely available and promoted, which will likely require policy and health system shifts away from target-based incentives and towards women-centered care. Concurrently, there is a need to shift the longstanding norms that sustain the cycle of early marriage, early in marriage fertility, and then sterilization once desired number and sex of children is achieved, something that gender-equity-focused family planning programs involving both men and women show promise in addressing [30–32]. This multilevel approach, involving government, policy makers, and society will be key in supporting the shift towards woman-centered contraceptive service provision that meets the needs of all Indian women.

## Supplementary Information

**Additional file 1.** Demand for family planning satisfied (DFPS) and share of each type of contraceptive being used across Indian states and union territories, 2015-16.

## Data Availability

NFHS-4 data is publicly available at https://dhsprogram.com/data/available-datasets.cfm

## Abbreviations

DFPS: Demand for family planning satisfied
IUD: Intrauterine device
LARC: Long-acting reversible contraceptive
NFHS: National Family and Health Survey
PSU: Primary sampling unit
SARC: Short-acting reversible contraceptive
SWPER: Survey-based Women’s emPoWerment Index
