# Supplementary material for: Modern contraceptive use among women in need of family planning in India: an analysis of the inequalities related to the mix of methods used
Source: Reprod Health. 2021 Aug 21;18:173. doi: 10.1186/s12978-021-01220-w (PMC8379729; doi:10.1186/s12978-021-01220-w)
Supplement: Supplementary file 1 — Additional file 1. Demand for family planning satisfied (DFPS) and share of each type of contraceptive being used across Indian states and union territories, 2015-16. [file 12978_2021_1220_MOESM1_ESM.docx]

Supplementary Table 1. Demand for family planning satisfied (DFPS) among partnered women aged 15-49 years and the share of each type of contraceptive being used among the users across Indian states and union territories, 2015-16 (N=339,540).

| **States** | **DFPS (95% CI)** | **Share of short-acting reversible methods**  **(95% CI)** | **Share of long-acting reversible methods**  **(95% CI)** | **Share of permanent methods**  **(95% CI)** |
| --- | --- | --- | --- | --- |
| Andaman and Nicobar Islands | 73.0 (68.8 - 76.8) | 13.2 (10.1 - 17.0) | 4.4 (3.1 - 6.1) | 82.5 (78.1 - 86.1) |
| Andhra Pradesh | 93.6 (92.8 - 94.3) | 0.5 (0.3 - 0.8) | 0.3 (0.2 - 0.5) | 99.2 (98.8 - 99.4) |
| Arunachal Pradesh | 49.9 (47.3 - 52.5) | 44.7 (41.5 - 47.9) | 12.9 (11.3 - 14.7) | 42.4 (39.3 - 45.6) |
| Assam | 55.2 (53.8 - 56.6) | 67.7 (65.6 - 69.8) | 6.0 (5.4 - 6.7) | 26.3 (24.3 - 28.3) |
| Bihar | 51.4 (50.0 - 52.8) | 8.6 (7.9 - 9.4) | 2.1 (1.8 - 2.5) | 89.2 (88.3 - 90.1) |
| Chandigarh | 72.5 (68.0 - 76.6) | 53.1 (45.9 - 60.2) | 9.3 (6.3 - 13.5) | 37.6 (30.9 - 44.8) |
| Chhattisgarh | 78.6 (77.6 - 79.7) | 10.5 (9.5 - 11.5) | 2.9 (2.4 - 3.5) | 86.6 (85.4 - 87.8) |
| Dadra And Nagar Haveli | 66.0 (60.4 - 71.2) | 12.8 (7.8 - 20.4) | 3.6 (1.6 - 8.0) | 83.6 (74.5 - 89.9) |
| Daman And Diu | 60.5 (53.6 - 67.0) | 10.9 (7.4 - 15.8) | 7.6 (4.6 - 12.3) | 81.5 (75.3 - 86.4) |
| Goa | 56.5 (48.9 - 63.7) | 30.6 (22.9 - 39.6) | 3.7 (1.7 - 7.7) | 65.7 (56.1 - 74.1) |
| Gujarat | 67.4 (66.0 - 68.9) | 14.7 (13.3 - 16.2) | 7.1 (6.1 - 8.2) | 78.3 (76.3 - 80.1) |
| Haryana | 81.0 (79.6 - 82.4) | 25.1 (23.8 - 26.5) | 9.6 (8.9 - 10.5) | 65.3 (63.7 - 66.8) |
| Himachal Pradesh | 71.7 (69.5 - 73.7) | 27.5 (25.4 - 29.7) | 1.6 (1.2 - 2.3) | 70.9 (68.6 - 73.1) |
| Jammu And Kashmir | 66.1 (64.4 - 67.9) | 40.1 (38.1 - 42.1) | 6.2 (5.3 - 7.2) | 53.7 (51.7 - 55.7) |
| Jharkhand | 63.4 (62.1 - 64.8) | 13.5 (12.4 - 14.6) | 2.6 (2.2 - 3.0) | 83.9 (82.7 - 85.1) |
| Karnataka | 82.4 (81.0 - 83.8) | 3.5 (2.7 - 4.6) | 1.6 (1.3 - 2.1) | 94.8 (93.7 - 95.8) |
| Kerala | 75.3 (73.5 - 77.0) | 5.6 (4.8 - 6.5) | 3.2 (2.6 - 3.9) | 91.2 (90.1 - 92.2) |
| Lakshadweep | 33.6 (27.7 - 40.0) | 27.2 (18.3 - 38.4) | 4.2 (1.8 - 9.8) | 68.6 (57.1 - 78.2) |
| Madhya Pradesh | 77.9 (77.1 - 78.7) | 12.7 (11.9 - 13.5) | 1.1 (0.9 - 1.2) | 86.2 (85.4 - 87.0) |
| Maharashtra | 83.9 (82.9 - 84.9) | 15.6 (14.5 - 16.7) | 2.6 (2.2 - 3.1) | 81.8 (80.5 - 83.0) |
| Manipur | 23.6 (22.1 - 25.2) | 45.3 (41.8 - 48.8) | 29.5 (26.3 - 32.8) | 25.3 (22.3 - 28.5) |
| Meghalaya | 48.1 (44.5 - 51.7) | 62.0 (56.0 - 67.7) | 9.6 (7.8 - 11.9) | 28.3 (23.4 - 33.8) |
| Mizoram | 63.8 (61.2 - 66.2) | 41.1 (36.8 - 45.6) | 9.5 (7.6 - 11.8) | 49.4 (45.5 - 53.3) |
| Nagaland | 43.4 (41.0 - 45.7) | 25.8 (22.1 - 29.9) | 31.4 (27.8 - 35.3) | 42.8 (38.9 - 46.7) |
| Delhi | 69.5 (66.7 - 72.1) | 47.5 (44.4 - 50.6) | 11.2 (9.6 - 13.0) | 41.3 (38.4 - 44.2) |
| Odisha | 63.7 (62.5 - 64.9) | 34.7 (33.1 - 36.2) | 2.5 (2.1 - 2.9) | 62.9 (61.3 - 64.5) |
| Puducherry | 87.1 (83.5 - 90.0) | 2.1 (1.3 - 3.2) | 4.2 (2.7 - 6.3) | 93.8 (91.4 - 95.5) |
| Punjab | 80.9 (79.6 - 82.1) | 32.4 (30.9 - 34.0) | 10.2 (9.3 - 11.2) | 57.4 (55.8 - 59.0) |
| Rajasthan | 74.2 (73.4 - 75.1) | 21.1 (20.1 - 22.1) | 2.3 (2.0 - 2.6) | 76.6 (75.5 - 77.6) |
| Sikkim | 67.1 (64.2 - 70.0) | 40.6 (37.1 - 44.1) | 13.7 (11.6 - 16.0) | 45.8 (42.4 - 49.1) |
| Tamil Nadu | 83.0 (82.0 - 83.9) | 2.4 (2.1 - 2.8) | 3.6 (3.1 - 4.1) | 94.0 (93.4 - 94.6) |
| Tripura | 57.2 (54.4 - 60.0) | 66.1 (63.5 - 68.7) | 1.4 (0.8 - 2.2) | 32.5 (30.0 - 35.1) |
| Uttar Pradesh | 49.8 (49.0 - 50.5) | 41.3 (40.1 - 42.5) | 3.7 (3.4 - 4.0) | 55.0 (53.7 - 56.2) |
| Uttarakhand | 71.5 (69.9 - 73.0) | 39.7 (37.2 - 42.2) | 3.3 (2.8 - 4.0) | 57.0 (54.5 - 59.5) |
| West Bengal | 72.4 (70.9 - 73.8) | 46.2 (44.0 - 48.3) | 2.1 (1.7 - 2.5) | 51.8 (49.6 - 53.9) |
| Telangana | 88.1 (86.7 - 89.4) | 1.5 (1.1 - 2.1) | 0.6 (0.3 - 1.2) | 97.9 (97.1 - 98.4) |
